# Pyorrhœa Alveolaris

**Published:** 1892-08

**Authors:** H. J. Burkhardt

**Affiliations:** Batavia, N. Y.


ARTICLE VII.

### PYORRHŒA ALVEOLARIS.

BY H. J. BURKHARDT, D. D. S., BATAVIA, N. Y.

Read at the Twenty-fourth Annual Meeting of the Eighth District Dental Society, held in Buffalo, April 19 and 20, 1892.

In presenting a paper on this subject, I feel as though an apology was due, because I am unable to offer you anything especially new. My purpose is briefly to narrate experiences with this disease while, with my brother, in charge of the practice of the Dansville Sanatorium, and to give you the observations I have made since. I have some statistics which I intended presenting, but am unable to do so from lack of time in which properly to tabulate them. As you are well aware, people who spend any considerable amount of time at institutions of the kind mentioned must, of necessity, at least start in with a full purse, and as a rule they are above the average in intelligence, investigations as to hereditary influence in the various diseases are more easy and reliable than in most cases.

I will not burthen you by a recital of the many and various theories of eminent practitioners as to the etiology of pyorrhœa alveolaris. Our literature is quite full, and yet no general theory has been accepted. Local, constitutional and bacterial theories, have been almost exclusively discussed of late. The latter has not received the attention the question merits. The advocates of local and constitutional causes are nearly equally divided.

That pyorrhœa alveolaris is a local expression of a constitutional disease, in a large majority of cases, I can prove most conclusively by my investigations. I have seen and had charge of one family of three generations, each being unmistakable cases of pyorrhœa, and within a year three families, two generations of which were in the same condition. Notwithstanding all this, I have yet some faith in local conditions as a cause in some instances. Nearly all of those who came under my notice were suffering from rheumatism, gout, local or general catarrh, dyspepsia, liver or kidney disease, or intemperance. By far the large majority were those of dyspepsia, and they of all others were most difficult to treat. Rheumatism and the various kidney diseases belong to the same category. Those of lymphatic temperament and scrofulous diathesis were practically incurable. A most marked peculiarity of the dpspeptic was the age, number of teeth affected, and scant discharge from the gums in extreme cases.

Middle life is the time pyorrhœa usually selects for an attack, and in a large percentage of cases in which dyspepsia was the exciting cause, the average age was about thirty. Right and left superior sixth and twelfth year molars and inferior central incisors were almost invariably the afflicted members, and why these particular teeth were the ones selected I should be pleased to have some one explain. There were many peculiar features observed, characteristic of the usual systemic disorder, an enumeration of which, at this time, would consume too much of your patience.

You are all familiar with the diagnosis of these cases. Where there is little tartar present and little inflammation, the prognosis is unfavorable; but where there is an abundance of tartar, a fairly safe prognosis can be made. Of course the general health plays a very important part in the conclusions arrived at. In the treatment, authorities do not widely differ. My plan is first thoroughly to remove all tartar, carious and necrosed bone, which can not always be done at the first sitting on account of profuse hemorrhage where many teeth are involved, and when there is general hypertrophy of the gum tissue. Amputation of the root of a tooth having more than one root I consider good practice, as the remaining roots are usually abundantly able properly to support the tooth. Just here let me contradict the assertion so often made in conventions, that a pulpless tooth is not liable to be affected by pyorrhœa. About the worst cases I ever saw were those of pulpless teeth, and as a general thing they could not be saved. I did not take the patient's word in regard to the canal having been filled. In several cases I had performed the operation myself, which seemingly was a success. The pus pockets should be evacuated by the injection of peroxide of hydrogen, and where there is any considerable amount of congestion, free lancing should be resorted to.

Where the teeth of one or both jaws are affected, I am a firm believer in the heroic treatment, as advocated by Dr. Riggs. The next consideration should be fastening of the loose teeth, and right here there is a great diversity of opinion as to the right kind of an appliance. Swaged cusps of copper, connection by means of gold fillings, ligating with floss silk or gilling twine, and metal bands, have all been used. The swaged caps have been discarded by me on account of the time required to construct, the difficulty of obtaining a correct articulation, and the offensive taste. Connecting by means of gold fillings I consider impracticable, except in a few isolated cases. The dirty, pestilence-breeding floss and gilling twine I have no use for. My preference is for a band of either gold or platinum, 35 gauge and a quarter of an inch wide, made large enough to encircle all the teeth affected, and where there are only a few afflicted, one or two healthy teeth on each side. Wherever it is possible the rubber dam is applied, and if that can not be conveniently done, napkins are resorted to and the teeth thoroughly dried. Oxy-phosphate of zinc is then thinly mixed and spread or smeared over the labial and palatine surfaces, and the band slipped up about midway between the gum margin and occluding surface. Next, fine binding wire of silver or platinum is slipped through the gum margin, from the labial to the palatine surface, brought between the teeth near the incisive edge, and back to the gum, twisted, and the slack taken up. This is repeated in each space, and when the last one is reached you have all of the teeth firmly bandaged. Care should be taken to allow the patient to close the mouth frequently during the process of wiring, so that loose teeth are not drawn out of position. With the aid of the burnisher, a few blasts of hot air and the removal of surplus cement, the case is completed. This gives you a firm, strong, clean bandage, quickly constructed and applied, and comfortable for the wearer. Local treatment should be given every day for a few weeks. As a solvent, pure sulphuric acid has been very successful in my hands. For the gums I use tincture of iodine, salicylic and tannic acids, and a wash composed largely of hydronapthol, have proved very efficient. Where the secretions of the mouth are offensive, five or six grains of chloride of zinc to an ounce of water, used as a gargle, is very beneficial. Remedies must be frequently changed to meet the varying conditions.

When it comes to constitutional treatment, I always try to work in harmony with the family physicican. Cathartics, tonics and alteratives are decidedly useful. If the disease is due to an enfeebled condition of the body, disgestive stimulants and plenty of exercise in the open air are indicated. If it is due to any particular cause, such as rheumatism, dyspepsia, etc., strict attention should be given to proper constitutional treatment to correct the exciting cause. Sponge grafting I have tried, but not with any appreciable good result. Thorough cleanliness should be insisted upon, and the mouth kept in as nearly an aseptic condition as possible.

Just a word in conclusion as to the application of crown and bridge work in mouths of pyorrhœal patients. I am a thorough believer and an advocate of crowns and bridges in the right place, but this is not in mouths where there is any considerable recession of the gums or indications of pyorrhœa. I can speak feelingly on this phase of the question, because of numerous funerals of my own and others. Within two years I have removed several bridges and crowns where the work had been well done. Last fall a gentleman came to me with a bridge in the lower jaw, attached to a canine, second bicuspid, and twelfth year molar, so loose that I removed the whole business with my fingers without the least trouble. The work had been beautifully done by a competent dentist, but the mistake was in the selection of the mouth. This is only one of numerous cases. I thoroughly believe that no tooth in a mouth where pyorrhœa is present should be made to assist in supporting an appliance of any kind, or to do more than the work intended for it. I have several bridges in mouths with a predisposition to pyorrhœa which apparently are all right, but I have little faith in their permanence.—*Dental Pract. and Advertiser.*

